# Aging in place in urban and rural contexts—Voices of community nurses from Austria

**DOI:** 10.3389/fpubh.2026.1869649

**Published:** 2026-10-22

**Authors:** Tatjana Fischer, Anita Sackl, Karl Moder

**Affiliations:** 1 Department of Landscape, Water and Infrastructure, Institute of Spatial Planning, Environmental Planning and Land Rearrangement, BOKU University, Vienna, Austria; 2 Department for Health Professions and Long-Term Care, Austrian National Public Health Institute, Vienna, Austria; 3 Department of Natural Sciences and Sustainable Resources, Institute of Statistics, BOKU University, Vienna, Austria

**Keywords:** Austria, community health, health determinants framework, healthy aging, lived experience of community nurses, online survey, place-based information

## Abstract

The present study explores the place-based knowledge on “aging in place” of community nurses (CNs) in Austria to gain insights in the suitability of urban and rural municipalities as places for residence at old age. An online survey was conducted at the end of 2024. Records of 23 CNs out of 273 registered CNs were included. The information was coded using the health determinants model by Dahlgren & Whitehead and statistically analyzed using Fisher's exact test and Cohen's Kappa for assessing information consistency. Due to the small sample size and the number of missing values the results are presented in a descriptive manner. It was found that 1. CNs working in rural municipalities tendentially identify older adults as a particularly vulnerable population group and 2. CNs working 20+ hours a week claim a lack of caring and caregiving relatives. Moreover, on the one hand CNs consider unsuitable living (housing) conditions and (risk of) poverty in old age as major challenges hampering a decent aging in place, on the other hand above all demographic change having considerable impact on day-to-day work of CNs in the future. Although the empirical findings are neither representative for Austria nor for different spatial types of municipalities the responses are in line with international literature and thus may serve as a starting point for an interdisciplinary debate between spatial planners and public health experts on metrics for assessing the age-friendliness of municipalities.

## 1 Introduction

### 1.1 Significance of topic

One of the key priorities for older people is the ability to live in their own four walls for as long as possible, thereby reflecting the fundamental need for autonomy and independent living (1, 2). Double aging is in this context a demographic trend characterized by an increase in both the absolute and relative proportion of older people within the total population, alongside an increase in the number of people of very old age. The likelihood of needing care increasing with age coupled with double-aging gives rise to the following goals with regard to policies concerning the maintenance and improvement of health of older adults: 1. enabling a self-determined life in the domestic environment, and 2. the aspiration to increase the number of healthy years of life (3, 4). Both goals are interlinked as the quality of the living environment, namely housing and neighborhood, influences health in that way they can either support or hamper the fulfillment of basic and social needs, and thus successfully carrying out activities of daily living (including physical activity and mobility, access to goods and services for daily needs, and the fulfillment of social, cultural and recreational needs) at home or outside the home (5, 6). This in turn can positively or negatively impact on the wellbeing and quality of life, and therefore also on the health of an (older) person. In addition, this also affects all individuals in their immediate social environment who provide informal support or care, among them relatives, neighbors, friends, social and healthcare workers. The built environment and being embedded in a safe and supportive social environment, thus influences life satisfaction and quality of life, and hence physical (activity) and mental (social inclusion/participation) health, and is therefore a key determinant of health (7, 8). The international literature on gerontology and nursing science agrees on this point and applies either the concept of person-environment fit (9) or the concept of aging in place (10) to describe the quality of old age or the aging process, taking into account the living environment or the built environment. Both concepts 1. emphasize, on the one hand, the importance of aligning the individual needs and demands of (older) adults with the quality of their living conditions (housing and environmental conditions) for a decent life and on the other hand point out the importance of measures in the event of a mismatch; and 2. address the need of a person for autonomy and health (11). For rural, sparsely populated communities or regions, for instance shortcomings in infrastructure supply (12) are reported to negatively influence aging in place, whereas for cities the decline of social networks as well as in infrastructure are key factors (13). In a global context, the significance of the type of space is also evident in urban-rural health disparities resulting from differences in access to infrastructure (14), as well as in the ability to continue to live well at home even in old age (15). As the feasibility of aging in place both in urban and rural municipalities is depending on the quality of housing, built environment and public spaces, available and accessible social infrastructure and leisure venues as well as public transport (16). Regarding equity and spatial justice in the context of ‘aging in place', findings from the Global South highlight significant inequalities when it comes to access to healthcare (17). Furthermore, disparities and inequalities exist in transport and mobility, particularly with regard to affordability (18), as well as limited mobility options arising from multi-generational households, resulting in a reliance to other family members for transportation needs (19). It should also be noted that the social welfare regime determines the framework conditions for facilitating or impeding aging equity within a certain country. This encompasses the state pension system as well as the provision and subsidization of infrastructure (above all housing, healthcare and nursing services). While social democratic systems, for example, prioritize the alleviation of social inequalities, the comparatively low level of state support in conservative systems leads to greater dependence among older people on their own families or their children (20).

Spatial planning and community nursing intersect within a Health-in-All-Policies approach, as both address key social and structural determinants of health. From a theoretical perspective, this linkage can be grounded in the nursing metaparadigm, particularly the dimension of environment, which conceptualizes health as shaped by physical, social and spatial contexts (21). At the same time, spatial planning decisions are recognized as key levers for shaping population health and reducing health inequities (22). Integrating this theoretically grounded nursing perspective into spatial planning is therefore essential for promoting equitable access to care and supporting age-friendly, resilient communities (23).

However, the built environment is rigid. That is why from a short and mid-term perspective aging in place primarily rests on the development of intra-family support and caregiving, reliable informal networks, and the availability of care professionals (24). But family structures are changing and the spatial separation of the family members' places of residence alters, respectively reduces the intra-family support and caregiving potential (15, 25, 26). As a result, the costs of professional support e.g., for people at risk of poverty in old age can pose an increasing challenge, as can social isolation and, subsequently, loneliness (27). Given the developments and reasons outlined above, community nursing, as a low-threshold service, is an important component of public health promotion (28). In Austria, the need for this new health-oriented service is underpinned by the fact that 1. Austria's population is aging – as of February 2026 there are 1.9 million people aged 65 and over living in Austria, which is approximately 21% of the total population (29) and 2. there are spatial disparities relating to both double-aging and population decline as well as to the provision of (social) infrastructure (30).

### 1.2 Motivation for the study and research questions

International empirical findings, showing the spatial-related enablers and barriers to aging in place, primarily originate from research conducted with old(er) adults (31). Qualitative research on the views of professionals such as social and community workers regarding the physical and symbolic components of aging in place regarding their relevance for independent living in old(er) age is also available (32). However, the place-based knowledge of community nurses could be of interest to spatial planners, as community health workers are familiar with the health- and infrastructure-related as well as the social needs of the local population in general, and of marginalized population groups in particular (33) as community nurses (CNs) are aware of their clients' living arrangements and social spaces (34). This presumably ‘unvarnished' insight into the lived environments and needs of older adults might help spatial planning consultants and political decision-makers to learn more about the space-health-care nexus – in other words, the connection between the built environment and the existence and nature of social networks (35, 36). Furthermore, this knowledge could enrich the discourse on (social) spatial justice (37, 38), the operationalisation of the Healthy Cities framework (39), as well as the effectiveness of spatial planning strategies aimed to mitigate any local and urban-rural disparities. However, in relation to the significance of the stakeholder landscape in spatial planning negotiations, there is as yet a lack of empirical evidence for assessing the benefits of involving community nurses – in terms of their advocate role for (vulnerable) older adults – with regard to developing a strategic knowledge base.

In Austria, a pilot project called Community Nursing was funded from 2022 to 2024 by EUGenerationNext (40) which aimed at promoting the wellbeing of individuals and communities and to address gaps in care by offering preventive, advisory and coordinating services provided directly to people (41). As CNs are outreach workers also in Austria and make preventive home visits (41), it is reasonable to assume that they are familiar with their clients' housing situations and living conditions (42). Furthermore, within the framework of the pilot project an accompanying training programme was provided to CNs to strengthen their personal and professional core competencies. CNs were asked to complete a Community Health Assessment which was based on Walking and Windshield Surveys (28, 43), two ways of exploring the municipalities by either walking, biking or by car. This is intended to provide initial insights into the municipalities' settlement structures, public spaces and infrastructure locations. 242 of the 2,092 Austrian municipalities took part in the Community Nursing pilot project, amongst them 200 rural municipalities (own calculations). These 242 municipalities were organized in 117 pilot projects. However, the project has not been followed up in all municipalities. As the project continued, it did so within different institutional frameworks (40).

The idea to explore aging in place from the perspective of community (health) nurses was born in June 2023 during a discussion with CNs following a talk given by the primary author at the lecture series for community nurses called “Maturity and Vulnerability in Old Age. How can Community Nursing contribute to quality of life?”, when a CN outlined the challenges in the municipality where she was working and explicitly expressed her interest in further discussions with spatial planners regarding the age-friendliness of municipalities. Due to a lack of empirical studies on aging in place at the local level for Austria in general, and from the perspective of social and healthcare professionals in particular, the primary author decided to carry out an independent study by conducting a standardized online survey with community nurses. The aim of this study was to assess the age-friendliness of municipalities in terms of not only the feasibility and quality of aging in place, but also in terms of the working conditions of the CNs, thereby highlighting any urban-rural differences. Organizational support was provided by the Austrian National Public Health Institute, especially by the secondary author.

This brief research report provides an overview of the empirical results addressing three research questions as follows:

1. What determines the age-friendliness of local authorities from the CNs' perspective?
2. Which spatial developments influence the CNs' day-to-day work?
3. What is the situation regarding the CNs' involvement in local planning?

### 1.3 Conceptual framework

As the aim of the study is to capture and describe both the local living conditions of older people and the working conditions of CNs from the CNs' perspective, the health determinants model by Dahlgren & Whitehead (8, 44) serves as a suitable operational model, as it encompasses both the spatial and relational aspects, which are addressed by both the concepts of person-environment fit and aging in place. Dahlgren's and Whitehead's model is therefore applicable in principle to any target group. Moreover, it provides the big picture of how spatial as well as relational aspects and health are intertwined at different (spatial) scales, thereby joining the concept of person-environment-fit with the concept of aging in place. The model describes that, in addition to a person's physiological and demographic characteristics and individual lifestyle factors, human health is impacted by housing conditions, social and community networks (family, neighborhood) as well as overarching socio-economic, cultural and environmental conditions, which in turn shape (individual) living and working conditions. For this study, this model was expanded to include the aspect of accessible infrastructure, which is a key priority of the WHO's Age-Friendly Cities Concept (45).

## 2 Material and methods

A cross-sectional quantitative study design was chosen.

### 2.1 Participant recruitment and online survey

To make the most efficient use of resources, it was decided to conduct an online survey using LimeSurvey. The invitation to participate in the survey was communicated by the Austrian National Public Health Institute through its monthly consultation sessions as well as via the CN newsletter. During the pilot phase, the newsletter was distributed to all 117 pilot projects, while designated representatives of the pilot projects participated in the consultation sessions. As of the date of the survey, 273 CNs were registered in Austria. The online survey was conducted between November and December 2024. This period at the end of the Community Nursing project was deliberately chosen to collect the feedback of as many CNs as possible, hoping that CNs with several years of experience will also participate in the survey. Prior to accessing the online questionnaire, CNs had to tick the participant information sheet to confirm their informed consent.

### 2.2 Operationalisation of the health determinants and questionnaire

By using an online survey, CNs were asked to share their perspective on aging in place as well as on the framework conditions of their day-to-day work. Based on the defined roles and competencies of community nurses in local authorities (46) (cf. Supplementary material S1) a questionnaire (cf. Supplementary material S2) consisting of 22 predominantly open-ended questions organized into six thematic sections was created. All health determinants as defined by Dahlgren & Whitehead (8, 44) – namely personal lifestyle, social and community networks, living and working conditions, as well as general conditions relating to the socio-economic, cultural and physical environment (hereinafter referred to as ‘general conditions') (Figure 1) – were addressed in thematic section 3, 4, and 5 of the questionnaire.

**Figure 1:**
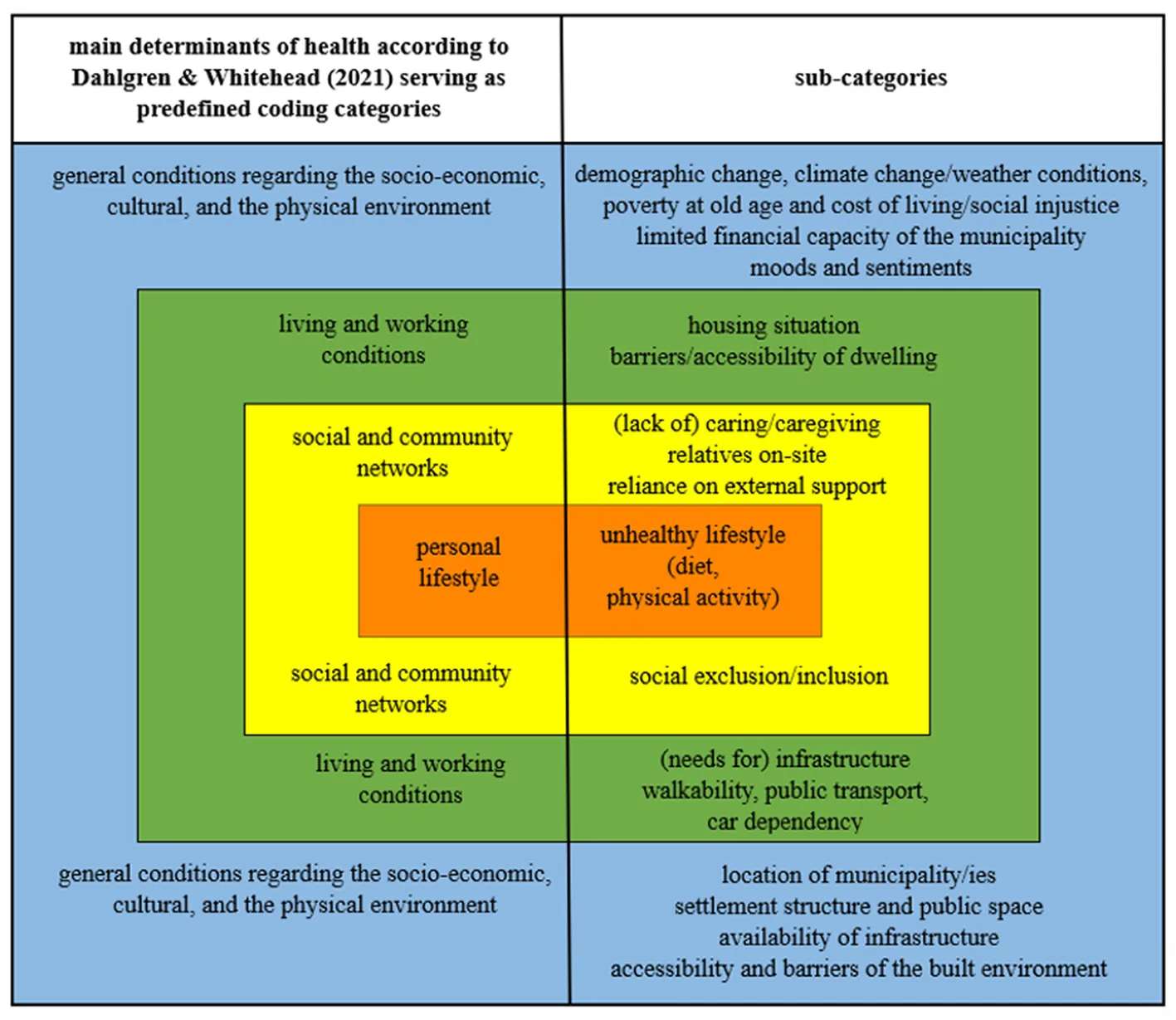
Coding frame and coding categories based on the health determinants model by Dahlgren & Whitehead (8, 44). The color scheme was inspired by the Austrian National Public Health Institute.

Thematic section 3, ‘Everyday observations' (questions no. 3/1–3/4), addresses municipal-specific challenges, which might have an impact on the fulfillment of the CNs' tasks and responsibilities, and which might also affect the vulnerability of the older clients living here. The questions included in this thematic section relate to the health determinants ‘living and working conditions' and ‘general conditions', which in turn pertain to both older clients and the CNs themselves.

Thematic section 4, “future trends” (questions no. 4/1 and 4/2), relates to the CNs' appraisal of the impact of future trends on the CN's working conditions and addresses both the health determinant “general conditions” and “social and community networks” in relation to the CNs' health and wellbeing.

Thematic section 5, ‘impressions of and wishes for the municipality' (questions no. 5/1 and 5/2), relates to the suitability or unsuitability of the municipality for a good life in old age and refers to all four health determinants outlined above in relation to healthy and comfortable aging of the (older) clients residing in the municipality.

Thematic section 2, ‘Communication and cooperation with local stakeholders' (questions no. 2/1–2/3), was aimed to obtain information on the CN's office location(s), their client groups, and the nature of the CN's engagement with local stakeholders, as well as the subject matters of the engagement.

To assess the CNs' role as stakeholders in spatial planning and development, thematic section 6, ‘Involvement in local planning and development processes' (questions no. 6/1–6/7), was created.

Furthermore, the following personal information (questions no. 1/1–1/4) was collected in thematic section 1 to identify any urban–rural differences, as well as the significance of the CN's experience and scope of employment on their response behavior and their assessment of the relevance of the individual health determinants. It was mandatory to answer questions no. 1/1–1/3. To protect the anonymity of the respondents, neither demographic information was collected nor were the names of the municipalities disclosed.

The structure of the questionnaire, and thus the chronological order of the thematic sections, was decided in dialogue with the secondary author of this brief research report, who also contributed to the phrasing of the questions to appeal to the respondents the most possible.

### 2.3 Coding process

To quantify the content of the open-ended questions relating to municipal-specific challenges impacting the CNs' day-to-day work (question no. 3/1) and to age-(un)friendliness of the municipality (question no. 5/2) the health determinants model by Dahlgren & Whitehead (8, 44) served as the overarching coding framework. Of the four ‘spheres' of the model, the following three served as predefined coding categories: ‘general conditions'; ‘living and working conditions'; and ‘social and community networks' (Figure 1). With regard to the operationalisation of the coding categories, we adopted the method of qualitative content analysis described by Boyatzis (47). Coding book examples are provided as Supplementary material S3. As only the primary author had access to the original data, independent double coding could not be performed.

### 2.4 Selection of records to be included in the statistical analysis

The statistical analysis should only include those data records: 1. which can be assigned to a spatial type of municipality on the basis of the real names of the municipalities (question no. 1/1); 2. for which all questions of the first half of the questionnaire are answered; and 3. in which older people are not referred to as clients (question no. 2/2).

### 2.5 Statistical analysis

#### 2.5.1 Hypotheses and measures

The coded responses to the open-ended questions were converted into the following subject-related statements:

1. Concerning the vulnerability of older adults (question no. 3/2):

- Older adults are considered one of many vulnerable groups.
- Older adults are considered particularly vulnerable compared to other client groups.
- The very old are considered particularly vulnerable compared to the other client groups.

2. Concerning the suitability of the municipality as a place of residence for older adults (or aging in place) (question no. 5/2):

- The municipality/-ies is/are a suitable place of residence for older adults.
- The municipality/-ies is/are not a suitable place of residence for older adults.
- The municipality/-ies is/are a suitable place of residence for older adults due to favorable general conditions.
- The municipality/-ies is/are not a suitable place of residence for older adults due to unfavorable general conditions.
- The municipality/-ies is/are a suitable place of residence for older adults due to favorable living conditions (infrastructure and mobility).
- The municipality/-ies is/are not a suitable place of residence for older adults due to unfavorable living conditions (infrastructure and mobility).
- The municipality/-ies is/are a suitable place of residence for older adults due to their social and community networks (e.g., availability of caring and caregiving relatives).
- The municipality/-ies is/are not a suitable place of residence for older adults due to their social and community networks (e.g., lack or absence of caring and caregiving relatives).

3. Concerning the municipality-specific challenges hampering the CNs' daily work (question no. 3/1):

- General conditions of the socio-economic, cultural and physical environment are regarded as challenging.
- The characteristics of social and community networks are regarded as challenging (e.g., lack or absence of caring and caregiving relatives).

4. Concerning the trends and framework conditions regarded relevant for the CN's daily work (question no. 4/1):

- Demographic change/aging, climate change and poverty at old age are equally important.
- Demographic change is crucial.
- Climate change is crucial.
- Poverty at old age is crucial.

5. Concerning the CNs' communication and cooperation with (local) stakeholders (question no. 2/3):

- CNs have contact to or are in dialogue with (local) politicians.
- CNs have contact to or are in dialogue with public authorities.
- CNs have contact to or are in dialogue with associations (incl. The Church).

It was assumed that the CN's perception and appraisal are related to the working experience, the level of employment as well as the spatial type of the municipality.

Additionally, it was assumed that the location of the CN's office is related to the communication with local stakeholders and decision-makers on social, health and spatial-related issues.

#### 2.5.2 Spatial type of municipality

This variable was coded as binary (urban/rural) based on the urban-rural typology of Statistics Austria (48) – namely, CNs working in urban municipalities (CNs_urban_) and CNs working in rural municipalities (CNs_rural_).

#### 2.5.3 Working experience in the municipality

Given the duration of the Community Nursing pilot project and assumed staff turnover, a categorization into ‘less than 1 year' and ‘1 year or more' was applied.

#### 2.5.4 Level of employment

Here, a classification in ‘up to 19 working hours a week' and ‘20+ working hours a week' was chosen. The threshold was set by default.

#### 2.5.5 Office location

This variable was classified as follows: located ‘in the municipal office or in another public building (e.g., town hall) in a central location', or ‘elsewhere'.

It was aimed to examine the associations between two categorical variables. An appropriate statistical method is the analysis of contingency tables. Statistical software SAS (version 9.4) was used. Due to the relatively small sample sizes, the commonly used approximate chi-square test was not suitable. For this reason, Fisher's exact test was used in the analysis. The significance level was set at 0.05. For all comparisons effect sizes are reported using Cramer's V and *p*-values – the latter serves as Supplementary information. 63 hypothesis tests were performed. No correction method was applied. All contingency tables (observed and expected frequencies) are provided in Supplementary material S4. To test the information consistency between the responses of CNs working in the same municipality, Cohen's Kappa (here: the “unweighted Kappa”) was used. A verbal classification of the results was carried out according to Landis & Koch (49).

## 3 Results

### 3.1 Dataset

#### 3.1.1 Sample size and response rate

41 out of a total of 273 CNs accessed the online survey; 31 provided real names of municipalities, 24 answered at least the first half of the questionnaire (up to and including question 3/4, cf. Supplementary material S2) – regardless of any missing entries in (sub)questions. 23 CNs identified older adults as their clients. Thus, 23 data records were included in the statistical analysis (Figure 2). Of these, 10 responses originated from CNs_urban_ and 13 from CNs_rural_, representing approximately one in four urban pilot projects and one in seven rural Community Nursing pilot projects.

**Figure 2:**
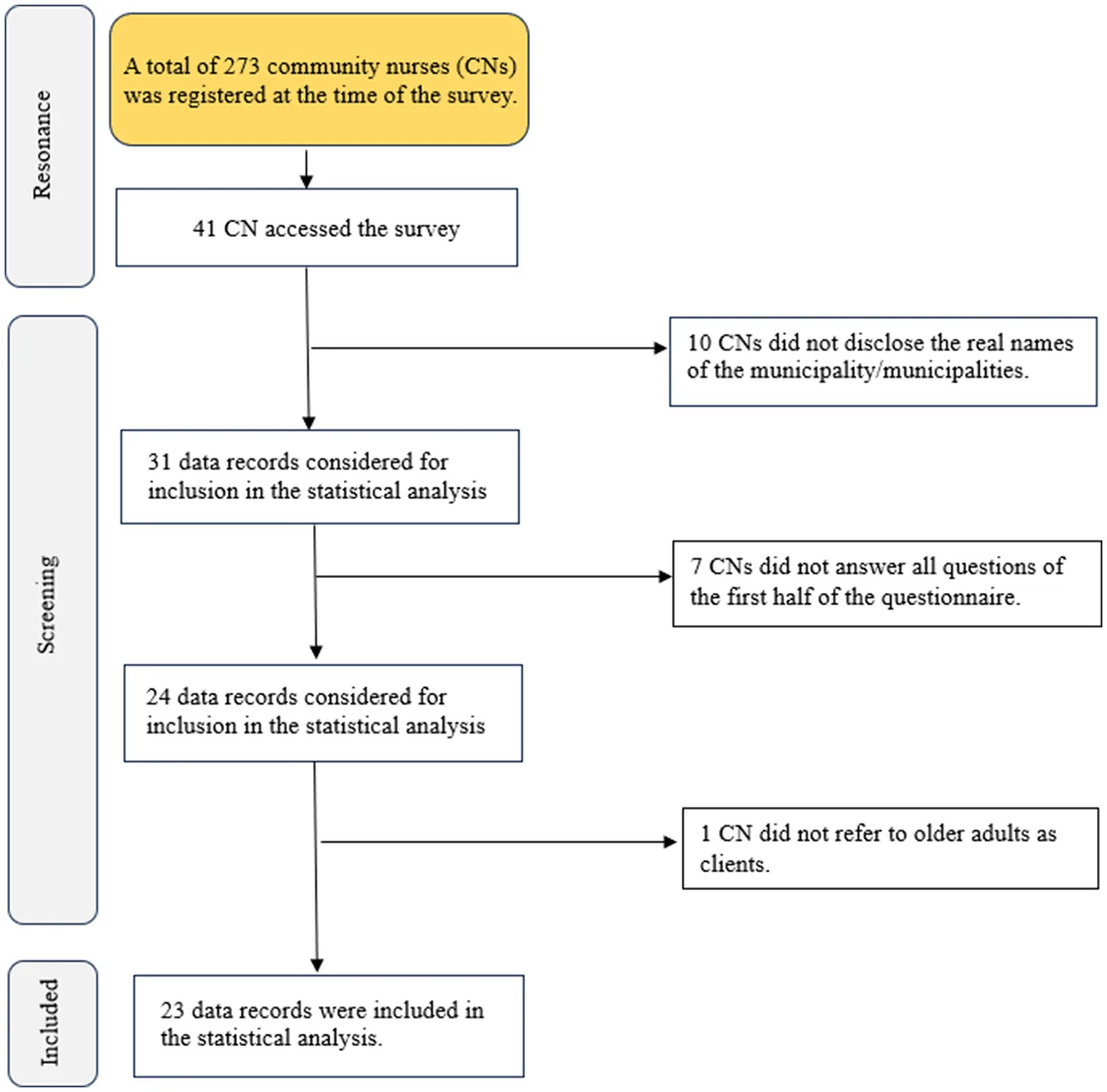
Flow chart on the CNs' response to the online survey.

#### 3.1.2 Frequency of responses

Neither the level of employment nor the spatial type of the municipality was found to be associated with the frequency of responses to the questions. The same applied for the working experience – except for question no. 6/1 (‘Who in the municipality or municipalities (e.g., local councilors, heads of associations) is interested in your spatial observations?'); (p-value = 0.0401) (cf. Supplementary material S5). However, it was found that 1. Questions appearing later in the questionnaire were answered less frequently and 2. CNs_rural_ tended to respond more frequently compared to CN_surban_ (Figure 3).

**Figure 3:**
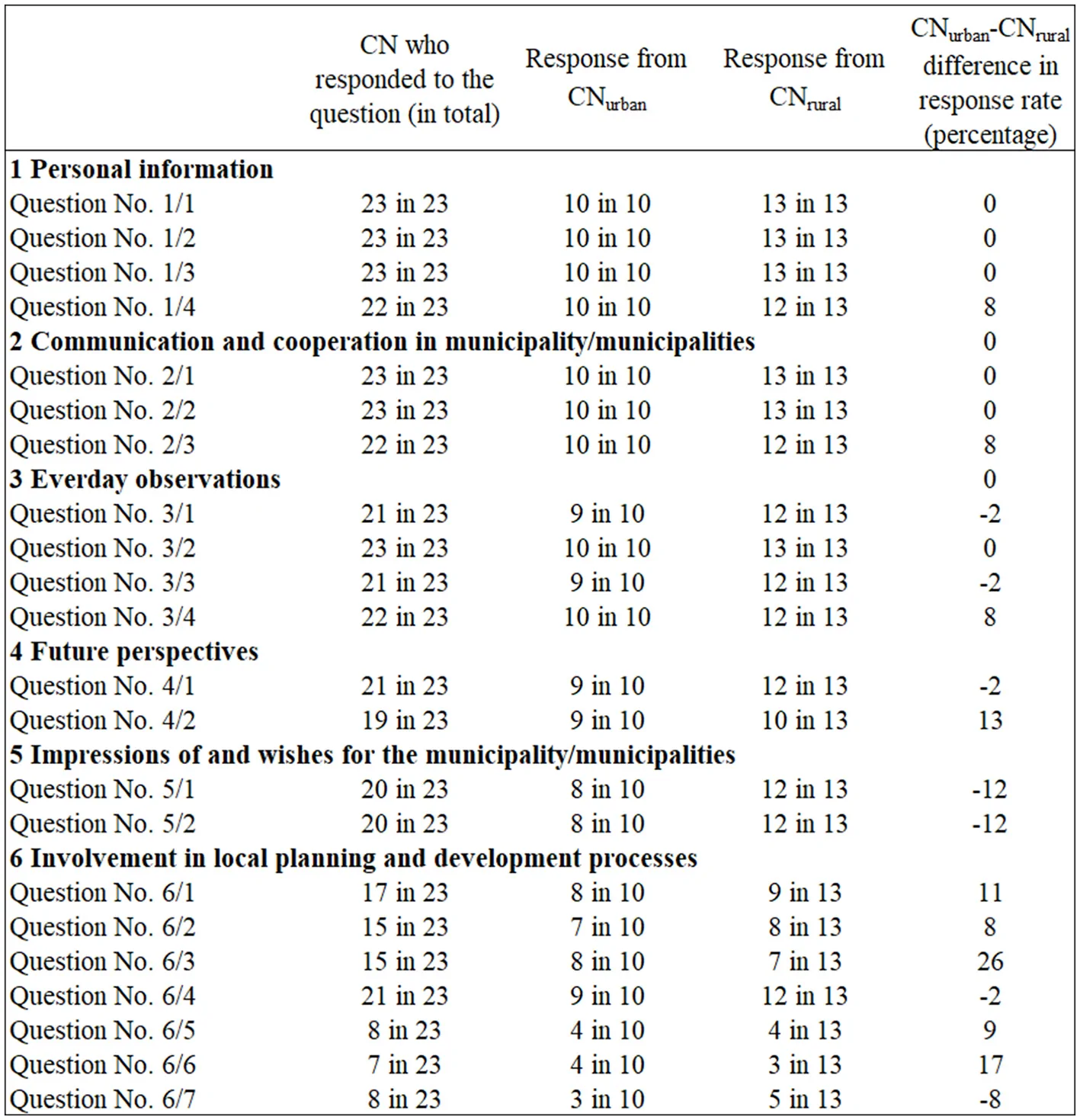
Frequency of responses by questions and urban-rural comparison.

Due to the small sample size and uncorrected multiple testing, the *p*-values presented below are of limited significance; they are used solely for exploratory description.

### 3.2 Vulnerability of older adults compared to other client groups

12 out of the 23 respondents reported older adults (including the very old) to be particularly vulnerable compared to other client groups; of these responding CNs 2 are CNs_urban_ and 10 CNs_rural_. Here, a certain tendency turned out between the spatial type of municipality and the perception of older adults (including the very old) being a vulnerable group compared to other client groups (*p*-value = 0.0123): Whereas CNs_urban_ identified older adults to be particularly vulnerable compared to other client groups significantly less frequently than expected, this was more frequent than expected among CN_rural_ (Figure 4). 10 out of 23 CNs considered older adults one of many vulnerable client groups. A certain tendency between the spatial type of municipality and this appraisal became evident (*p*-value = 0.0034). Whereas CNs_urban_ tendentially more often mentioned older adults one of many vulnerable client groups, the opposite is true for CNs_rural_, as shown by the expected value of 5.6522 compared to the observed value of 2. The recognition of vulnerable groups is not associating either with the working experience or with the level of employment (Figure 4).

**Figure 4:**
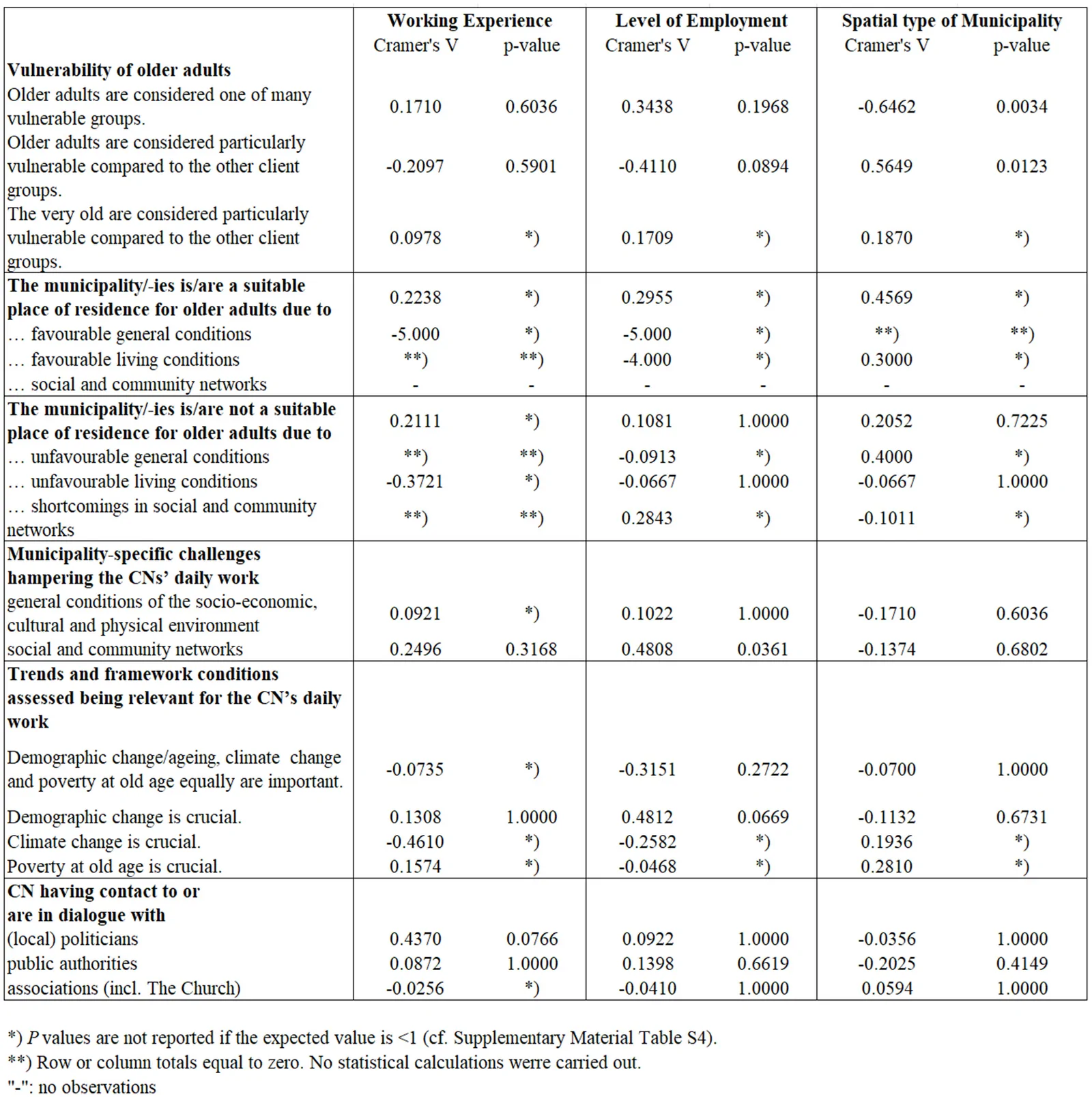
Effect Sizes. Cramer's V and *p*-values.

### 3.3 Appraisal of the age-friendliness of the municipality

9 (inclusive of 5 CNs_rural_) out of 23 CNs considered the municipality as a suitable place of residence at old(er) age, 5 CN (inclusive of 2 CN_rural_) did not. No association between the appraisal of the age-friendliness and the spatial type of municipality was detected in this sample. This also applied for the working experience and the level of employment (Figure 4).

Regarding the unsuitability of urban municipalities, social and community structures tend to be of greater importance than infrastructure provision and transport services. Shortcomings in these two areas tend to be key factors in rural municipalities.

### 3.4 Municipality-specific challenges faced in performing day-to-day work

CNs reported negative impacts on their day-to-day work caused by unsuitable ‘living (housing) situations' for aging (*n* = 16), car dependency (*n* = 12), and the lack of (caregiving) relatives (*n* = 12). Furthermore, weather conditions (*n* = 3) were also reported as a challenge. No associations between the spatial type of municipality and municipal-specific challenges were detected in this sample. The same applied to the working experience (Figure 4). The level of employment might be of certain relevance with regard to reporting the lack or rather absence of (caregiving) relatives as challenging in carrying out day-to-day work (*p*-value = 0.0361), because CNs who are working 20+ hours a week more often report the lack or absence of caring and caregiving relatives as a challenge (Figure 4).

### 3.5 Future trends and framework conditions relevant to CNs' day-to-day work

CNs prescribed relevance to demographic change/aging (*n* = 21), climate change/natural hazards (*n* = 20) and poverty in old age (*n* = 21) as future trends which will impact their work seriously. Neither any associations between the perception of the relevance of these trends and the spatial type of municipality nor any considerable associations with the working experience or the level of employment were detected in this sample (Figure 4). All contingency tables are provided in Supplementary material S4.

### 3.6 Communication with local stakeholders

CNs reported that they are engaged in conversation with local politicians (mayors) (*n* = 15), public authorities (e.g., heads of departments) (*n* = 13) as well as clubs and associations (including the Church) (*n* = 5). Two CNsrural were in dialogue with all three groups of stakeholders. No association between communication with certain stakeholders and the location of the CNs' offices was detected in this sample (cf. Supplementary material S6). It should be noted here that stakeholders with whom health-related and social issues are being discussed are neither interested necessarily in the CNs' spatial-related observations nor in their appraisal of the age-friendliness of the municipality.

### 3.7 Participation in local planning and municipal development

6 out of 23 CNs have already been involved in local planning, 6 CNs would like to do so in the future. Of those who have already been involved in local planning, 5 are CNs_rural_; of those who would like to be involved, 4 are CNs_urban_. 4 CNs (including 2 CNs_rural_) have already been involved in municipality development. Additionally, 5 CNs (including 2 CNs_rural_) were interested in getting involved. Only 1 CNs_rural_) was both involved in local planning and municipality development. 7 out of 23 CNs would like to share their place-based lived experiences (particularly regarding matters of accessibility) with public authorities or spatial planners.

## 4 Discussion and conclusion

To the best of the authors' knowledge, this study is the first attempt in the German-speaking countries to capture the local framework conditions of ‘aging in place' from the perspective of CNs, considering urban and rural differences and the spatial-related requirements for CNs. The study shows, that CNs_rural_ consider older people in their municipalities to be more vulnerable compared to other client groups, while CNs_urban_ identify older adults to be one of many vulnerable client groups. From the CNs' perspective, the lack of age-friendliness in urban municipalities is primarily determined by shortcomings in social and community structures, whilst in rural municipalities infrastructure provision and access to infrastructure are primary factors. The lack of (caregiving) relatives is perceived by CNs working 20+ hours a week a pivotal challenge in their day-to-day work with older clients. Housing and living situations of the older clients, as well as the CNs' car dependency are indicated as further key challenges. Concerning the future work as a CN, the respondents identify demographic aging and poverty in old age, as well as climate change and natural hazards, as issues of concern. CNs state that they barely share their lived experiences regarding spatial and non-spatial aspects of age-friendliness of the municipalities with local (political) decision makers or spatial planners. Specifically, only a few CNs are already involved in local planning and municipal development. However, due to the small sample size and high rate of missing data, all findings are preliminary and should not be generalized.

### 4.1 Limitations of the study

This study is purely exploratory and hypothesis-generating.

The survey was conducted during a phase of organizational uncertainty (completion of the pilot phase and transition into regular programmes). Furthermore, several evaluations of the Community Nursing project have been conducted during its pilot phase. This may have led to a saturation regarding participation in surveys (50), resulting in low participation. As the survey link was sent to the contact persons of the 117 pilot municipalities asking them to forward it to the CNs, the number of CNs who received the survey link cannot be verified. These conditions may have contributed to non-response bias. Since data on geographical distribution and workplace characteristics of the overall CN population in Austria is not available a selection bias cannot be ruled out. This may also result in a self-selection bias caused by the low participation of CNs.

Regarding statistical analysis, it should be noted that 1. No correction methods were applied and 2. The *p*-values represent uncorrected explanatory indicators and therefore are of limited significance, the empirical findings do not constitute confirmatory evidence. This is due to the small sample size and uncorrected multiple testing. So, the *p*-values are used solely for exploratory description. Consequently, this study does not claim to be representative or generalisable. Moreover, it proved impossible to assess the subjectivity of the CNs' responses. Nevertheless, in three cases a moderate-to-substantial agreement was observed in three cases (Kappa values 0.6099, 0.7249 and 0.6477).

Regarding the content quality of the results and the reproducibility of the study, it should be noted that coding – i.e., the assignment of answers to specific health determinants – played a key role and was taken very seriously in terms of methodology. As the coding of the responses was carried out solely by the primary author, the potential for multidisciplinary reflection on the raw empirical data may not have been fully utilized (51). Strengthening methodological rigor, through larger samples, qualitative interviews, or inter-coder reliability, would have enhanced credibility. Given that no triangulation or validation using mixed methods were performed, the results are exploratory and cannot be generalized.

Given the small sample size of both, CNs_urban_ and CNs_rural_, and the high number of missing values (Supplementary Table S3), the suitability of the municipalities as suitable places of residence for older adults cannot be attributed to specific health determinants, which also makes it difficult to compare urban and rural municipalities in this regard. Instead, the results are exploratory and descriptive in nature and can be used to generate hypotheses addressing questions concerning the correlations between aging in place and the built environment, as well as the relevance of physical and settlement structures and influencing factors affecting the work and future development of the CNs' workforce in urban and rural areas. Furthermore, due to the low response rate, the findings are limited in their generalisability to Austrian CNs.

As no automated, georeferenced data, namely indicators on gendered caregiving burdens, infrastructure and services, and socio-economic vulnerabilities which would allow for a spatial archetype related comparison of aging in place are available for Austria at the municipal level, it was impossible to interpret the reported lived experiences of community nurses in an analytical manner.

### 4.2 Comparison to existing knowledge

The CNs provided descriptions on general trends and developments – above all demographic aging and poverty at old age –, which are not related to specific spatial types of municipalities and – in their opinion – having a considerable impact on aging in place and their day-to-day work as a CN. These empirical findings mirror the relevance of demographic aging which implies a growing number of older adults suffering from multimorbidity, chronic diseases and complex needs for (health) care (52), which in turn requires CNs to develop complex plans for care provision, time and resource management, as well as counseling for patients and their relatives (52). Particularly in rural (alpine) regions, long distances, deficiencies in public transport, unfavorable housing conditions and, moreover, extreme weather conditions cause difficulties in delivering (health)care (53, 54). Additionally, gaps in care can arise due to the absence of family members, meaning that CN must take on additional tasks such as mobility and medication management (55, 56). The respondents' statements regarding the availability of supportive and caregiving relatives are of paramount importance, as they can be helpful for assessing the consequences that might result, such as conflicts between work and family life, increased stress and a reduced ability to identify gaps in home care at an early stage (57, 58). Furthermore, the availability and degree of support within the family might influence the long-term demand for CNs or perhaps even complicate the work as a CN. It would therefore be interesting to investigate to what extent CNs are willing to continue working in their familiar municipalities.

Moreover, the present study provides an insight into the awareness of CNs for the consequences of climate change (heatwaves or floods) for the health of older adults. This finding suggests that CNs ought to be able to identify acute impacts on health and coordinate preventive measures, which requires interprofessional collaboration (59). The latter follows from the fact that demographic aging and climate-related threats are overlapping which in turn increases the complexity of care planning, the burden on care staff, and the demands on preventive measures and crisis management. Therefore, the opportunity to age in place is supported by low-threshold healthcare, accessible infrastructure and transport, age-friendly housing and a supportive social environment (5), as well as well-functioning local amenities and digital infrastructure.

Furthermore, the respondents draw attention to the relevance of accessibility issues such as low population density, scattered settlements and long distances to essential services and the limited resources and capacities of communities and public authorities to support aging populations. International literature has already addressed these issues (60, 61). The CNs surveyed in this study are aware of housing issues such as housing insecurity amongst older adults, as well as the challenges involved in adapting housing to accommodate changing physical needs. These are aspects that research on aging and the built environment has already addressed (62, 63), emphasizing the intertwining with the income situation of older adults, as well as with constrained public finances that further limit support for older adults (60, 61, 64). These social inequalities, coupled with physical barriers (in both rural and urban environments), further exacerbate (small-scale) disparities in terms of older people's participation and social inclusion – and thus their ability to age in place, even in cities –, which are two aspects that the WHO counts amongst the eight core elements of an age-friendly city (65). By linking the empirical findings from the present study on locally relevant trends (particularly demographic aging), the description of infrastructure-related care provision structures and social and community structures (particularly social networks that vary from person to person, and the absence of family carers) with the structure of the built environment and general developments such as increasing staff shortages in the social and healthcare sectors, for Austria it can be cautiously concluded the following: a successful and decent aging in place, both in rural and peripheral municipalities with low population densities as well as in cities and towns may become more and more difficult for single older adults, older adults who have reduced mobility or are immobile and/or who are at risk of poverty. Thus, it is worthwhile to further explore this topic in collaboration with community health workers. Firstly, by linking illness trajectories with housing situations as part of a health impact assessment, healthcare professionals can clearly illustrate the severity of the real-life circumstances faced by old(er) adults to decision-makers responsible for local and regional planning (66); secondly, they can contribute to stimulating a more critical discussion in spatial planning about the opportunities and challenges of ‘aging in place' – at the local level by reflecting on the added value of mixed-use settlement structures, and at the regional level by evaluating key indicators relating to infrastructure provision and location planning. This is because it is assumed that cross-sectoral cooperation can truly help all parties involved to recognize the necessities and challenges of incorporating health-related objectives into their planning instruments. As far as spatial planning is concerned, this would primarily refer to the further development of the existing building stock (67). For example, the insights gained from this study could be incorporated into tools for the long-term assessment of the age-friendliness of settlement structures. Even though the present study for Austria shows that the respondents fulfill their function as information hubs on social and health issues (46), the reasons why local stakeholders do not show interest in the place-based knowledge of CNs and why CNs are not heavily involved in local planning could not be derived and therefore should be subject to further research.

### 4.3 Closing remark

This brief research report presents the opinions on the subject of ‘aging in place' expressed by approximately 8 % of the community nurses working in Austrian at the time of the online survey. The community nurses identified a need for action in the areas of housing (accessibility), mobility and access to infrastructure (public transport), and social networks (inclusion/participation) – issues that are in line both with the WHO's Age-Friendly Cities and Communities framework and referring to the SDGs, in particular with SDG 3 (good health and wellbeing) and SDG 11 (sustainable cities and communities).

## Data Availability

The original contributions presented in the study are included in the article/Supplementary material, further inquiries can be directed to the corresponding author.
