# Supplementary material for: Aging in place in urban and rural contexts—Voices of community nurses from Austria
Source: Front Public Health. 2026 Oct 22;14:1869649. doi: 10.3389/fpubh.2026.1869649 (PMC13735538; doi:10.3389/fpubh.2026.1869649)
Supplement: Supplementary file 1 [file Data_Sheet_1.pdf]

SUPPLEMENTARY MATERIAL S1. Roles and competencies of community nurses

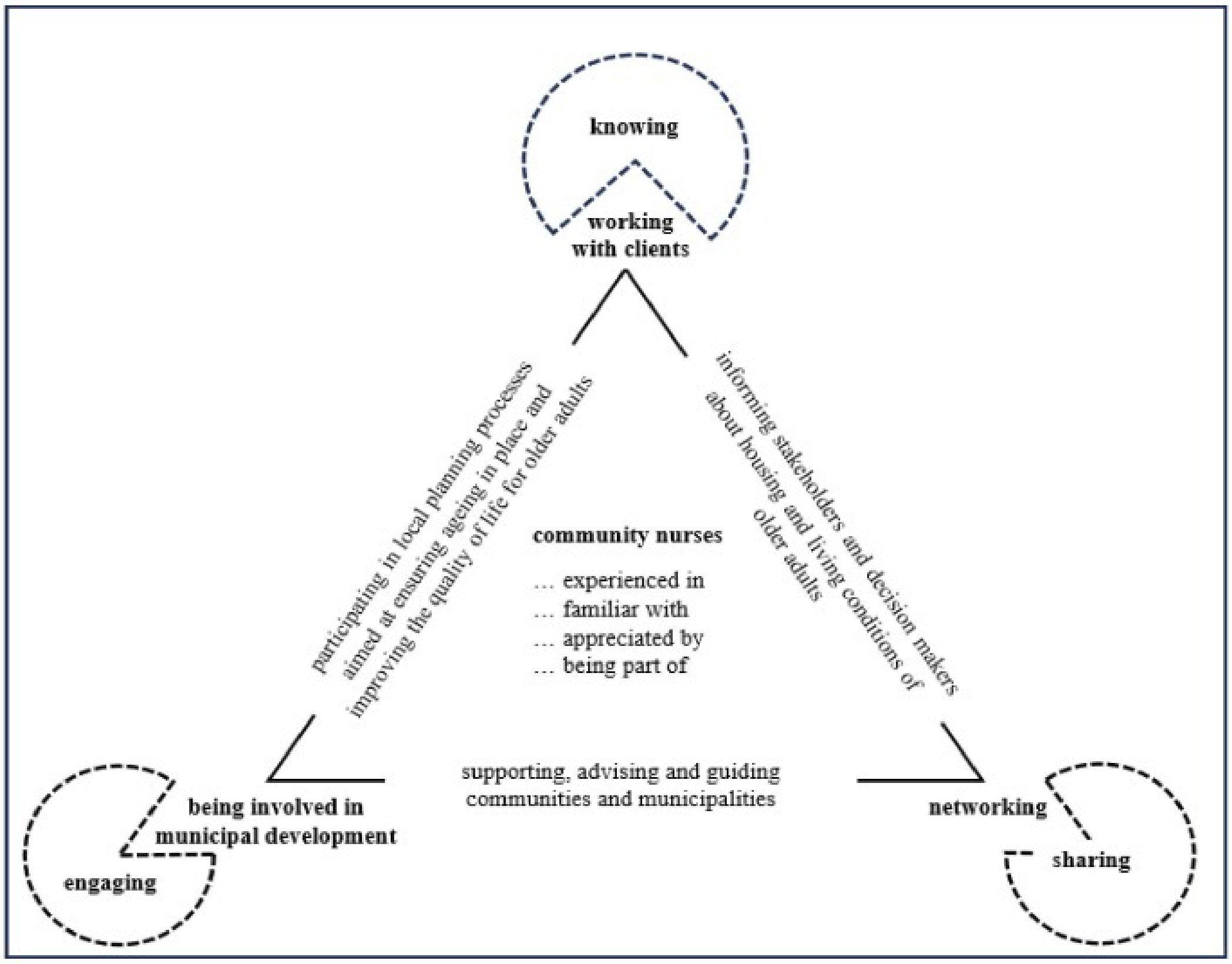

ALT TEXT SUPPLEMENTARY MATERIAL S1: a triangle showing the roles and competences of community nurses in Austria particularly related to knowledge exchange, networking and the presumed added value of involving community nurses as stakeholders in local planning

SUPPLEMENTARY MATERIAL S2. The questionnaire (translated into English)

Thematic section	Question No.	Wording of question	Type of question
1<br>Personal information	**1/1**	**I am currently working as a community nurse in the following municipality/municipalities …**	open-ended
	**1/2**	**For how long (number of months) have you been working in the respective municipality/municipalities?**	open-ended
	**1/3**	**I am available in the following municipality/municipalities for the … number of hours per week.**	open-ended
	1/4	That's how I became a community nurse in the municipality/municipalities:	open-ended
2<br>Communication and cooperation stakeholders	**2/1**	**My office(s) in the municipality/municipalities is/are located in a) in the municipal office, b) in another public building in central location, c) in a building in the outskirts, d) in a building near the industrial area, e) elsewhere. Please select and comment on your satisfaction with the location of the office(s).**	closed-ended multiple choice with space for further comments
	**2/2**	**My work as a community nurse involves providing service to the following groups of people …**	open-ended
	**2/3**	**Who are the representatives of the municipality/municipalities you are in dialogue with regarding what kinds of social and health-related issues?**	open-ended
3<br>Everyday observations	**3/1**	**In fulfilling my tasks and responsibilities, I am facing the following municipality-specific challenges:**<br>**a) long distances/car dependency, b) weather conditions, c) lack of (caregiving) relatives available on site, d) unsuitable living conditions for everyday living/life in old age, e) other. Please select the applicable options and comment on them.**	closed-ended multiple choice with space for further comments
	**3/2**	**In the municipality/municipalities I am considering the following clients/groups of persons vulnerable: a) children, b) young adults, c) women, d) older persons, e) the very old, f) persons with migration background who have recently moved into the municipality, g) other, because ...**	closed-ended multiple choice with space for further comments
	3/3	What kinds of clients' vulnerabilities in the municipality/municipalities particularly are affecting you and for what reason?	open-ended
	3/4	Based on your lived experience: How do your clients react to specific issues the municipality/municipalities (e.g., lack of provision of goods, lack of socio-medical services, lack of sense of community or rather lack of like-minded or similar affected persons, feeling of not being listened to etc.) and changing framework conditions (e.g., climate change and heat or natural hazards)?	open-ended
4<br>Future Trends	**4/1**	**In the future, the following trends and framework conditions in the municipality/ municipalities will make my work more challenging (1 = strongly disagree, 2 = somewhat disagree, 3 = agree, 4 = strongly agree, 5 = strongly agree): a) demographic change/ageing, b) climate change and the impacts of heat and natural hazards, c) poverty at old age, d) other.**	closed-ended multiple choice

	**4/2**	**Please outline on why the trends and framework conditions identified relevant will make your work more challenging in the municipality/municipalities (in the future).**	open-ended
5 Impressions of and wishes for the municipality/ municipalities	5/1	When I first started working here, the first thing I noticed was...	open-ended
	**5/2**	**The suitability of the municipality/ies as (a) place(s) of residence for my clients, I rate as follows: If possible, please provide an appraisal for particular target groups (e.g., young people, persons at older age, persons who are poor or at risk of poverty, persons with or without a migration background who have moved in more recently, persons living alone, etc.).**	open-ended
6 Involvement in local planning and municipal development	**6/1**	**Who in the municipality/municipalities (e.g., political municipal representatives, heads of associations) is interested in your placed-based observations (e.g., long distance trips for daily supply, challenges regarding the provision of goods and services) and is addressing you specifically in this regard? Please specify the spatial perceptions.**	open-ended
	6/2	ad 6/1 How would you rationalise this interest?	open-ended
	6/3	ad 6/2 On what occasions are you asked about your spatial observations in the municipality/municipalities?	open-ended
	**6/4**	**Do spatial planners ask you for your input in the context of elaborating local planning instruments (development concepts, zoning, building development plans)? If yes, regarding what?**	open-ended
	**6/5**	**Are you involved in any municipal development processes (Local Agenda 21, LEADER projects, ...)? If so, which ones?**	open-ended
	**6/6**	**If you have not been yet involved in any local spatial development processes, though you would like to be: Which ones would that be, and how would you ideally prefer to be involved?**	open-ended
	**6/7**	**With whom would you like to share your place-based knowledge about the municipality/municipalities as well as their challenges and potentials, and for what purpose?**	open-ended

ALT TEXT SUPPLEMENTARY MATERIAL S2: a figure presenting the questionnaire titled ‘Community Nurses’ Place-Related Knowledge in Austria’. The questions analysed for this brief research report are highlighted in bold

SUPPLEMENTARY MATERIAL S3. Coding book examples

Original response	Sub-category	Category
"… Older adults with only a low pension struggle to cover their cost of living..." (ID 10)<br><br>"The location [of the municipality] is very good; public transport connections are very good; there is a shortage of affordable public housing, very little affordable housing, and limited accessibility in the municipality." (ID 15)<br><br>"I think xxx is a very good municipality for all groups of people; its social orientation is very strong." (ID 20)	limited financial capacity of the municipality<br><br>location of municipality<br>limited financial capacity of the municipality<br><br>mood and sentiments	general conditions relating to the socio-economic environment<br><br>general conditions relating to the physical environment<br><br>general conditions relating to the cultural environment
"Older adults, people who are materially deprived or at risk of poverty, as well as people who are living alone: by improving public transport, we can alleviate the fear of isolation for those who are no longer mobile." (ID 6)<br><br>"There are many services available. The problem of limited transportation should be addressed in future by a new social transport service." (ID 12)<br><br>"Basically good, but we need more facilities such as day centres or centres providing respite and intermediate care to support informal caregivers, perhaps a community centre where people can meet for free." (ID 18)<br><br>"Yes, the expansion of the nursing home offering short-term care would be very important." (ID 28)	walkability, public transport, car dependency<br><br>(needs) for infrastructure<br>walkability, public transport, car dependency<br><br>(needs for) infrastructure<br><br>(needs for) infrastructure	living and working conditions
"Older adults, people who are materially deprived or at risk of poverty, as well as people who are living alone: by improving public transport links, we can alleviate the fear of isolation for those who are no longer mobile." (ID 6)	social exclusion/inclusion	social and community networks

ALT TEXT SUPPLEMENTARY MATERIAL S3: a figure presenting coding book examples

SUPPLEMENTARY MATERIAL S4. Contingency Tables

	**Working Experience**				**Level of Employment**				**Spatial Type of Municipality**			
	**less than one year**		**one year or more**		**up to 19 working hours per week**		**20+ working hours per week**		**urban**		**rural**	
	observed	expected	observed	expected	observed	expected	observed	expected	observed	expected	observed	expected
**Vulnerability of older adults**												
Older adults are considered one of many vulnerable groups.	*p-value* = 0.6036				*p-value* = 0.1968				*p-value* = 0.0034			
yes	1	17 391	9	8.2609	2	3.913	8	6.087	8	4.3478	2	5.6522
no	3	22 609	10	10.739	7	5.087	6	7.913	2	5.6522	11	7.3478
Older adults are considered particularly vulnerable compared to other client groups.	*p-value* = 0.5901				*p-value* = 0.0894				*p-value* = 0.0123			
yes	3	2 087	9	9.913	7	4.6957	5	7.3043	2	5.2174	10	6.7826
no	1	1 913	10	9.087	2	4.3043	9	6.6957	8	4.7826	3	6.2174
The very old are considered particularly vulnerable compared to the other client groups.												
yes	0	0.1739	1	0.8261	0	0.3913	1	0.6087	0	0.4348	1	0.5652
no	4	3.8261	18	18.174	9	8.6087	13	13.391	10	9.5652	12	12.435
**The municipality/-ies is/are a suitable place of residence for older adults.**[a)]												
yes	1	1.5652	8	7.4348	3	3.5217	6	5.4783	5	3.913	4	5.087
no	0	0.3478	2	1.6522	0	0.7826	2	1.2174	2	0.8696	0	1.1304

The municipality/-ies is/are a suitable place of residence for older adults due to favourable general conditions.												
yes	1	0.6667	1	1.3333	1	0.6667	1	1.3333	0	0	2	0
no	0	0.3333	1	0.6667	0	0.3333	1	0.6667	0	0	1	1
The municipality/-ies is/are a suitable place of residence for older adults due to favourable living conditions (infrastructure and mobility).												
yes	0	0	5	5	2	1.4286	3	3.5714	1	1.4286	4	3.5714
no	0	0	2	2	0	0.5714	2	1.4286	1	0.5714	1	1.4286
The municipality/-ies is/are a suitable place of residence for older adults due to their social and community networks (e.g. availability of caring and caregiving relatives).												
yes	-	-	-	-	-	-	-	-	-	-	-	-
no	-	-	-	-	-	-	-	-	-	-	-	-
**The municipality/-ies is/are not a suitable place of residence for older adults.**[b)]					*p-value* = 1.0000				*p-value* = 0.7225			
yes	0	0.6957	4	3.3043	2	1.5652	2	2.4348	2	1.7391	2	2.2609
no	1	0.8696	4	4.1304	2	1.9565	3	3.0435	3	2.1739	2	2.8261
The municipality/-ies is/are not a suitable place of residence for older adults due to unfavourable general conditions.												
yes	0	0	2	2	1	0.8571	1	1.1429	0	0.5714	2	1.4286

no	0	0	5	5	2	2.1429	3	2.8571	2	1.4286	3	3.5714
The municipality/-ies is/are not a suitable place of residence for older adults due to unfavourable living conditions (infrastructure and mobility).						*p-value* = 1.0000				*p-value* = 1.0000		
yes	1	0.3571	4	4.6429	2	1.7857	3	3.2143	2	1.7857	3	3.2143
no	0	0.6429	9	8.3571	3	3.2143	6	5.7857	3	3.2143	6	5.7857
The municipality/-ies is/are not a suitable place of residence for older adults due to their social and community networks (e.g. lack or absence of caring and caregiving relatives).												
yes	0	0	2	2	0	0.6154	2	1.3846	1	0.7692	1	1.2308
no	0	0	11	11	4	3.3846	7	7.6154	4	4.2308	7	6.7692
**Municipality-specific challenges hampering the CNs' daily work**												
General conditions of the socio-economic, cultural and physical environment are regarded as challenging.						*p-value* = 1.0000				*P-value* = 0.6036		
yes	3	3.3043	16	15.696	7	7.4348	12	11.565	9	8.2609	10	10.739
no	1	0.6957	3	3.3043	2	1.5652	2	2.4348	1	1.7391	3	2.2609
The characteristics of social and community networks are regarded as challenging (e.g. lack or absence of caring and caregiving relatives).		*p-value* = 0.3168				*p-value* = 0.0361				*p-value* = 0.6802		
yes	1	2.087	11	9.913	2	4.6957	10	7.3043	6	5.2174	6	6.7826
no	3	1.913	8	9.087	7	4.3043	4	6.6957	4	4.7826	7	6.2174

**Trends and framework conditions regarded relevant for the CN's daily work**												
Demographic change/ageing, climate change and poverty at old age equally are important.						*p-value* = 0.2722				*p-value* = 1.0000		
yes	1	0.7619	3	3.2381	3	1.7143	1	2.2857	2	1.7143	2	2.2857
no	3	3.2381	14	13.762	6	7.2857	11	9.7143	7	7.2857	10	9.7143
Demographic change is crucial.		*p-value* = 1.0000				*p-value* = 0.0669				*p-value* = 0.6731		
yes	1	1.5238	7	6.4762	1	3.4286	7	4.5714	4	3.4286	4	4.5714
no	3	2.4762	10	10.524	8	5.5714	5	7.4286	5	5.5714	8	7.4286
Climate change is crucial.												
yes	1	0.1905	0	0.8095	1	0.4286	0	0.5714	0	0.4286	1	0.5714
no	3	3.8095	17	16.19	8	8.5714	12	11.429	9	8.5714	11	11.429
Poverty at old age is crucial.												
yes	0	0.381	2	1.619	1	0.8571	1	1.1429	0	0.8571	2	1.1429
no	4	3.619	15	15.381	8	8.1429	11	10.857	9	8.1429	10	10.857
**CN having contact to or are in dialogue with …**												
(local) politicians		*p-value* = 0.0766				*p-value* = 1.0000				*p-value* = 1.0000		
yes	1	2.7273	14	12.273	5	5.4545	10	9.5455	7	6.8182	8	8.1818
no	3	1.2727	4	5.7273	3	2.5455	4	4.4545	3	3.1818	4	3.8182
public authorities		*p-value* = 1.0000				*p-value* = 0.6619				*p-value* = 0.4149		
yes	2	2.3636	11	10.636	4	4.7273	9	8.2727	7	5.9091	6	7.0909
no	2	1.6364	7	7.3636	4	3.2727	5	5.7273	3	4.0909	6	4.9091
associations (incl. The Church)						*p-value* = 1.0000				*p-value* = 1.0000		
yes	1	0.9091	4	4.0909	2	1.8182	3	3.1818	2	2.2727	3	2.7273
no	3	3.0909	14	13.909	6	6.1818	11	10.818	8	7.7273	9	9.2727

Note:

[a)]no precise statements referring to variable "spatial type of municipality" (CN_{urban}: 3 observed frequencies, 5.2174 expected frequencies; CN_{rural}: 9 observed frequencies, 6.7826 expected frequencies);
no precise statements referring to variable "level of employment" (less than 20 working hours a week: 6 observed frequencies, 4.6957 expected frequencies;
20+ working hours a week: 6 observed frequencies, 7.3043 expected frequencies)
no precise statements referring to variable "working experience" (less than one year: 3 observed frequencies, 2.087 expected frequencies;
one year and more: 9 observed frequencies, 9.913 expected frequencies)
"-" = no observations
[b)] no precise statements referring to variable "spatial type of municipality" (CN_{urban}: 5 observed frequencies, 6.087 expected frequencies; CN_{rural}: 9 observed frequencies, 7.913 expected frequencies);
no precise statements referring to variable "level of employment" (less than 20 working hours a week: 5 observed frequencies, 5.4783 expected frequencies;
20+ working hours a week: 9 observed frequencies, 8.5217 expected frequencies)
no precise statements referring to variable "working experience" (less than one year: 3 observed frequencies, 2.4348 expected frequencies;
one year and more: 11 observed frequencies, 11.565 expected frequencies)

ALT TEXT SUPPLEMENTARY MATERIAL S4: a table presenting all contingency tables – namely observed and expected frequencies – inclusive of notes referring to missing values or rather unprecise statements

SUPPLEMENTARY MATERIAL S5. Number of responses by question

	Working Experience					Level of Employment					Spatial type of Municipality				
	less than one year		one year ore more			up to 19 working hours		20+ working hours per			urban		rural		
	response	no response	response	no response	p-value	response	no response	response	no response	p-value	response	no response	response	no response	p-value
**1 Personal information**															
Question No. 1/4	4	0	18	1	1.0000	9	0	13	1	1.0000	10	0	12	1	1.0000
**2 Communication and cooperation in municipality/municipalities**															
Question No. 2/1	4	0	19	0		9	0	14	0		10	0	13	0	
Question No. 2/2	4	0	19	0		9	0	14	0		10	0	13	0	
Question No. 2/3	4	0	18	1	1.0000	8	1	14	0	0.3913	10	0	12	1	1.0000
**3 Everday observations**															
Question No. 3/1	3	1	18	1	0.3241	8	1	13	1	1.0000	9	1	12	1	1.0000
Question No. 3/2	4	0	19	0		9	0	14	0		10	0	13	0	
Question No. 3/3	3	1	18	1	0.3241	8	1	13	1	1.0000	9	1	12	1	1.0000
Question No. 3/4	4	0	18	1	1.0000	9	0	13	1	1.0000	10	0	12	1	1.0000
**4 Future perspectives**															
Question No. 4/1	4	0	18	1	1.0000	9	0	13	1	1.0000	10	0	12	1	1.0000
Question No. 4/2	4	0	15	4	1.0000	8	1	11	3	1.0000	9	1	10	3	0.6036
**5 Impressions of and wishes for the municipality / municipalities**															
Question No. 5/1	3	1	17	2	0.1629	8	1	12	2	1.0000	8	2	12	1	0.5596
Question No. 5/2	2	2	18	1	0.0666	8	1	12	2	1.0000	8	2	12	1	0.5596
**6 Involvement in local planning and development processes**															
Question No. 6/1	1	3	16	3	0.0401	5	4	12	2	0.1616	8	2	9	4	0.6600
Question No. 6/2	1	3	14	5	0.1028	5	4	10	4	0.6570	7	3	8	5	1.0000
Question No. 6/3	2	2	13	6	0.5889	5	4	10	4	0.6570	8	2	7	6	0.3788
Question No. 6/4	3	1	18	1	0.3241	8	1	13	1	1.0000	9	1	12	1	1.0000
Question No. 6/5	0	4	8	11	0.2569	2	7	6	8	0.3998	4	6	4	9	0.6850
Question No. 6/6	0	4	7	12	0.2727	3	6	4	10	1.0000	4	6	3	10	0.6500
Question No. 6/7	0	4	8	11	0.2569	4	5	4	10	0.6570	3	7	5	8	1.0000

Note: For questions where no p-value is reported, Fisher's exact test cannot be calculated because one column in the contingency table is empty (questions 2/1 and 2/2). As a result, the matrix becomes singular during the calculation.

ALT TEXT SUPPLEMENTARY MATERIAL S5: a table presenting a contingency table referring to the number of responses by question considering working experience, level of employment and spatial type of municipality

SUPPLEMENTARY MATERIAL S6. Contingency table “Locations of Community Nurses’ offices in the Municipalities”

	**Locations of Community Nurses' offices in the Municipalities**			
	**in the municipal office/town hall or in another building in central location**		**elsewhere**	
	observed	expected	observed	expected
**CN maintains contact to and dialogue with (local) politicians.**				
yes	15	14.318	0	0.6818
no	6	6.6818	1	0.3182
**CN maintains contact to and dialogue with public authorities.**				
yes	13	12.409	0	0.5909
no	8	8.5909	1	0.4091
**CN maintains contact to and dialogue with associations (incl. The Church).**				
yes	5	4.7727	0	0.2273
no	16	16.227	1	0.7727

ALT TEXT SUPPLEMENTARY MATERIAL S6: a table presenting a contingency table referring to the locations of the community nurses’ offices and the community nurses’ communication with local stakeholders
